# Bile acid-axis dysregulation in MASLD/MASH progression: from metabolic mismatch to inflammatory-fibrotic remodeling

**DOI:** 10.3389/fmed.2026.1912000

**Published:** 2026-08-18

**Authors:** Yang Du, Lin Qi, Yunze Shi, Xingyuan Huang, Xiaopei Shi, Mingfeng Liu, Ying Fang, Xiaoyang Hu

**Affiliations:** 1 Graduate School, Heilongjiang University of Chinese Medicine, Harbin, Heilongjiang, China; 2 Basic Medical College of Heilongjiang University of Chinese Medicine, Harbin, Heilongjiang, China

**Keywords:** bile acids, FXR, gut microbiota, intestinal barrier, liver fibrosis, MASH, MASLD, TGR5

## Abstract

Metabolic dysfunction-associated steatotic liver disease (MASLD) and metabolic dysfunction-associated steatohepatitis (MASH) arise from overlapping metabolic stress, gut-derived inflammatory input, immune activation, and fibrotic remodeling. Bile acids participate in cholesterol metabolism and lipid absorption, and they also regulate metabolic, intestinal barrier, immune, and inflammatory responses through farnesoid X receptor (FXR), G protein-coupled bile acid receptor 1 (TGR5/GPBAR1), fibroblast growth factor 19 (FGF19), and related signaling pathways. Accordingly, bile acids may constitute an important interface linking intrahepatic metabolic abnormalities with gut–liver inflammatory crosstalk, although many human findings remain associative and require cautious mechanistic interpretation. This review summarizes bile acid-axis dysregulation in MASLD/MASH, focusing on intrahepatic bile acid synthesis and transport, microbiota-mediated bile acid pool remodeling, intestinal barrier disruption, and the intrahepatic inflammatory-fibrotic niche. We propose that bile acid dysregulation should be interpreted beyond simple increases or decreases in total bile acid levels. Instead, it may reflect a systemic mismatch among bile acid pool composition, compartmental distribution, microbial transformation, and receptor-mediated signaling output. Future studies should integrate multi-compartment bile acid profiling with microbial enzyme activity, intestinal barrier assessment, and tissue-specific receptor signaling to support mechanism-based stratification, treatment-response monitoring, and rational combination strategies.

## 1 Introduction

Metabolic dysfunction-associated steatotic liver disease (MASLD) is currently one of the most common chronic liver diseases. It may progress from simple hepatic steatosis to metabolic dysfunction-associated steatohepatitis (MASH), liver fibrosis, cirrhosis, and hepatocellular carcinoma (1–3). The 2023 multisociety Delphi consensus promoted the nomenclature transition from non-alcoholic fatty liver disease/non-alcoholic steatohepatitis (NAFLD/NASH) to MASLD/MASH, emphasizing that the disease should be defined from the perspective of metabolic dysfunction rather than through the exclusionary term “non-alcoholic” (4). The 2024 EASL-EASD-EASO Clinical Practice Guidelines further placed MASLD within the framework of cardiometabolic risk management. This shift indicates that prevention and treatment should not be limited to intrahepatic fat content itself, but should also address dynamic changes in metabolic abnormalities, inflammatory activity, and fibrosis risk (5).

In recent years, the mechanistic understanding of MASLD progression to MASH has gradually shifted from a “fat accumulation model” to a “metabolic–inflammatory–fibrotic coupling model” (6–8). Increased fatty acid uptake, enhanced *de novo* lipogenesis, insufficient mitochondrial fatty acid oxidation, disturbed cholesterol homeostasis, and insulin resistance jointly promote the accumulation of lipotoxic molecules, including diacylglycerols, ceramides, free cholesterol, and saturated fatty acids. Lipotoxicity subsequently induces mitochondrial dysfunction, oxidative stress, endoplasmic reticulum stress, and cell death (7, 9). Injured hepatocytes release damage-associated molecular patterns (DAMPs), chemokines, and extracellular vesicles. These signals activate Kupffer cells, monocyte-derived macrophages, and hepatic stellate cells (HSCs), thereby creating a tissue microenvironment in which inflammation and fibrosis mutually amplify each other (7, 9).

Within this multilayered network, the bile acid axis has substantial value for mechanistic integration. Bile acids participate not only in cholesterol catabolism and lipid absorption, but also in glucose and lipid metabolism, feedback regulation of bile acid synthesis, intestinal barrier homeostasis, and immune and inflammatory regulation through the farnesoid X receptor (FXR), G protein-coupled bile acid receptor 1 (TGR5/GPBAR1), fibroblast growth factor 19 (FGF19), small heterodimer partner (SHP), and related signaling pathways (10–12). Bile acid signaling is inherently embedded in the enterohepatic circulation. Bile acids synthesized in the liver and secreted into the intestinal lumen can be deconjugated, dehydroxylated, oxidized/reduced, and epimerized by the gut microbiota, producing bile acid profiles that differ in hydrophobicity, receptor affinity, and effects on the intestinal barrier (13–15). When the intestinal barrier is impaired, bile acid metabolites, lipopolysaccharide (LPS), bacterial DNA, fungal components, and other microbial metabolites may continuously enter the liver through the portal vein. A lesion initially dominated by metabolic stress may therefore acquire a sustained source of inflammatory amplification (11, 16).

**Figure 1:**
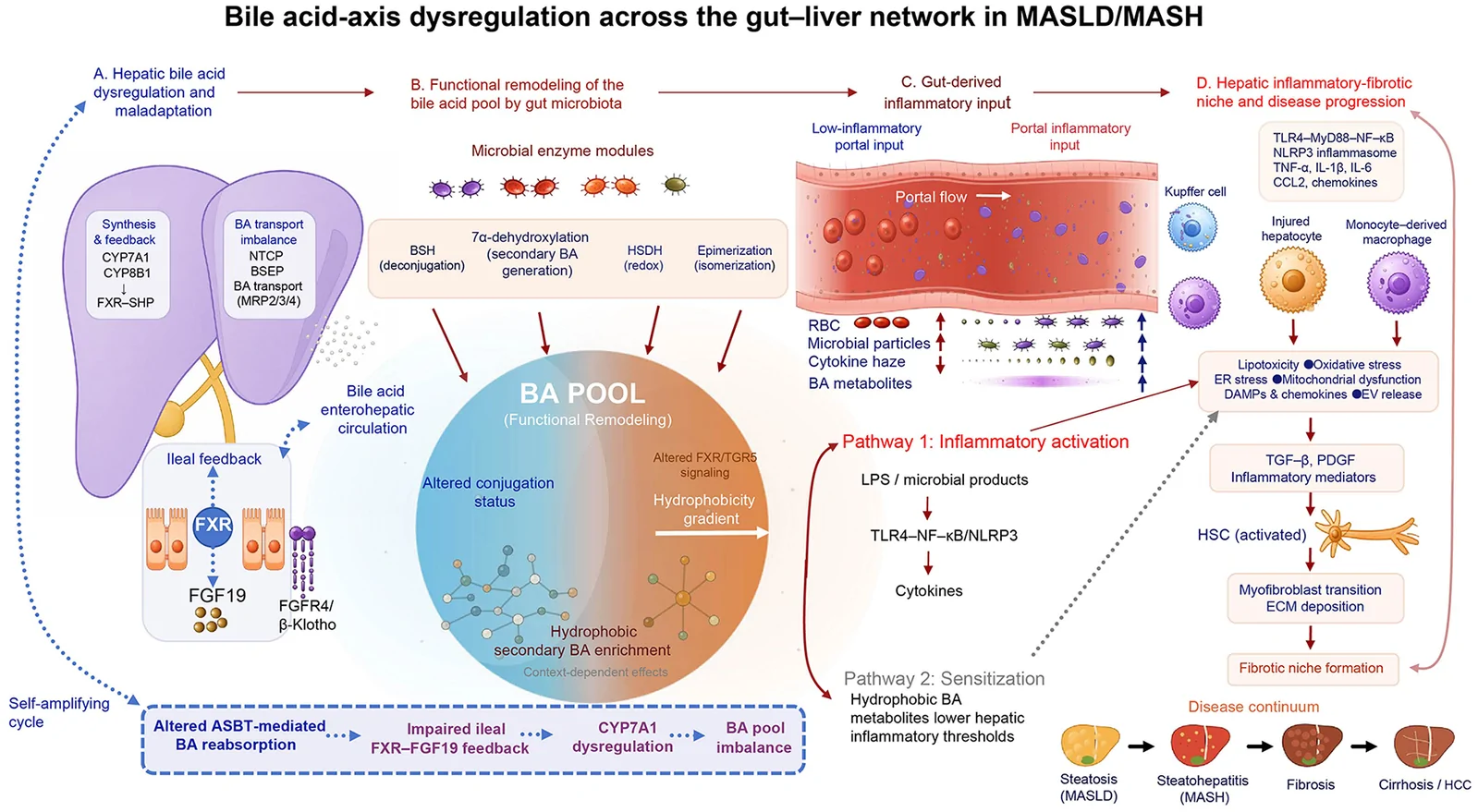
Evidence-stratified framework of bile acid-axis dysregulation across the gut–liver network in MASLD/MASH. The figure summarizes how hepatic bile acid maladaptation, microbiota-mediated bile acid pool remodeling, gut-derived inflammatory input, and intrahepatic inflammatory–fibrotic remodeling may interact during MASLD/MASH progression. Red solid arrows indicate established inflammatory–fibrotic pathways, including TLR4–MyD88–NF-κB/NLRP3 activation, inflammatory cytokine and chemokine signaling, hepatocellular injury, HSC activation, myofibroblast transition, extracellular matrix deposition, and fibrotic niche formation. Blue dashed arrows indicate emerging enterohepatic feedback mechanisms, including altered ASBT-mediated bile acid reabsorption, impaired ileal FXR–FGF19 feedback, CYP7A1 dysregulation, and bile acid pool imbalance. Gray dot-dash arrows indicate proposed hypothesis-generating links, particularly the sensitization model in which hydrophobic bile acid metabolites may lower hepatic inflammatory thresholds and facilitate disease progression. This framework distinguishes mechanisms with different levels of evidentiary support and does not imply equivalent causal certainty for all depicted interactions.

Recent reviews have increasingly discussed bile acid signaling, gut–liver microbiome crosstalk, and microbiota-derived metabolites in MASLD/MASH (17–19). Building on these studies, the novelty of the present review does not lie in proposing the gut–liver axis as an entirely new field, but in organizing the bile acid axis into a three-layer interpretive framework that links intrahepatic bile acid maladaptation, microbiota-mediated bile acid pool remodeling, intestinal barrier-dependent portal inflammatory input, and remodeling of the hepatic inflammatory-fibrotic niche. This review therefore does not treat the bile acid axis as a linear pathway centered on a single receptor or one class of bile acid molecules. Instead, it reconsiders this axis within the gut-liver network that may contribute to MASLD/MASH progression. The review is organized around three interconnected levels: upstream intrahepatic metabolic mismatch, an intermediate microbiota–bile acid–intestinal barrier interface, and downstream remodeling of the intrahepatic inflammatory–fibrotic niche. Particular emphasis is placed on the microbiota–bile acid–intestinal barrier interface because this interface may help explain how the intestinal bile acid pool is functionally reprogrammed by microbial activity and how luminal metabolic events may be converted into portal inflammatory input associated with intrahepatic inflammation and fibrotic responses (13, 16, 20). To facilitate interpretation of this integrative framework, Figure 1 summarizes a simplified directional model linking hepatic bile acid maladaptation, microbiota-mediated bile acid pool remodeling, barrier disruption with portal inflammatory input, and remodeling of the hepatic inflammatory–fibrotic niche. Solid arrows indicate the main directional framework and relatively well-supported pathways, whereas dashed arrows indicate emerging or context-dependent links.

## 2 Upstream events: bile acid homeostatic imbalance and intrahepatic metabolic reprogramming

### 2.1 Bile acid synthesis, transport, and FXR–FGF19 feedback

Bile acid homeostasis depends on coordinated regulation of synthesis, transport, intestinal transformation, and enterohepatic circulation; it cannot be attributed to a single enzymatic step (10, 21). Intrahepatic bile acid production is mainly controlled by key synthetic enzymes, such as cholesterol 7α-hydroxylase (CYP7A1) and sterol 12α-hydroxylase (CYP8B1), as well as by the FXR–SHP and FXR–FGF19 feedback axes. Bile acid circulation between the liver and intestine depends on multiple transporters. Sodium taurocholate cotransporting polypeptide (NTCP/SLC10A1) on the basolateral membrane of hepatocytes participates in the uptake of conjugated bile acids, whereas the bile salt export pump (BSEP/ABCB11) mediates canalicular bile acid excretion. Multidrug resistance-associated proteins, including MRP2/ABCC2, MRP3/ABCC3, and MRP4/ABCC4, regulate the efflux of bile acids and related organic anions. In the terminal ileum, the apical sodium-dependent bile acid transporter (ASBT/SLC10A2) mediates bile acid reabsorption, whereas organic solute transporter α/β (OSTα/OSTβ) participates in the transfer of bile acids from enterocytes into the portal circulation (10).

In MASLD/MASH, the significance of these synthetic, transport, and feedback systems extends beyond changes in intracellular bile acid burden within hepatocytes. These systems also reshape the bile acid substrate pool that enters the intestinal lumen and is subsequently processed by the microbiota. Once synthetic feedback, enterohepatic circulation efficiency, and microbial transformation become uncoupled, bile acid profiles in plasma, feces, portal blood, and liver tissue may shift in different directions (20, 22). Thus, the defining feature of bile acid dysregulation is not a simple increase or decrease in total bile acid amount, but a mismatch among bile acid pool composition, hydrophobicity, conjugation status, compartmental distribution, and receptor-mediated signaling output.

The FXR–FGF19 feedback axis is a key link between intestinal bile acid sensing and intrahepatic bile acid production. Activation of ileal FXR by bile acids induces FGF19 secretion. FGF19 then reaches the liver via the portal vein and acts on the fibroblast growth factor receptor 4 (FGFR4)/β-Klotho complex, thereby suppressing the CYP7A1-mediated classical bile acid synthesis pathway (10, 23). If ileal inflammation, microbial dysbiosis, or altered bile acid pool composition impairs ileal FXR activation, intrahepatic feedback regulation of bile acid synthesis may be weakened. Conversely, when liver injury or impaired bile acid efflux increases bile acids in the systemic circulation, plasma bile acid levels may primarily reflect abnormal hepatic clearance and spillover rather than enhanced intestinal feedback.

### 2.2 Bile acid signaling and intrahepatic metabolic adaptation

Early MASLD is not merely passive triglyceride accumulation. It results from the combined effects of increased fatty acid uptake, enhanced DNL, insufficient mitochondrial fatty acid oxidation, abnormal very-low-density lipoprotein (VLDL) export, disturbed cholesterol metabolism, and insulin resistance (7, 9, 24). The core driver of MASH progression is hepatocellular injury, inflammation, and fibrotic progression induced by toxic lipids and metabolic intermediates rather than fat content alone (25). Against this background, bile acid signaling occupies an important regulatory node in the metabolic network. The FXR–SHP axis can influence sterol regulatory element-binding protein 1c (SREBP-1c)-related lipogenic programs, intestinal FXR–FGF19 feedback can regulate bile acid production and cholesterol metabolism, and receptors such as TGR5 may participate in energy metabolism and inflammatory regulation (10).

Disturbed bile acid signaling should not be interpreted as an isolated cause of hepatic steatosis. Under conditions of nutritional excess and insulin resistance, a more plausible interpretation is that bile acid signaling mismatch weakens the liver's adaptive capacity to lipid influx, cholesterol burden, and oxidative stress. This process can amplify lipotoxicity, inflammatory injury, and subsequent fibrotic responses (26). The upstream significance of the bile acid axis therefore lies in shaping the substrate pool and signaling context for intestinal bile acid remodeling, rather than functioning as a closed intrahepatic metabolic pathway in isolation (27).

The composition of the bile acid pool also affects intrahepatic metabolic phenotypes. 12α-hydroxylated bile acids, non-12α-hydroxylated bile acids, conjugated bile acids, and unconjugated bile acids are not equivalent in receptor affinity, hydrophobicity, or cytotoxicity (27, 28). Some bile acid molecules may enhance FXR signaling and restrict bile acid synthesis, whereas others may aggravate hepatocellular injury indirectly through membrane perturbation, mitochondrial stress, or inflammatory receptors (27, 29). Measurement of total bile acids alone is therefore insufficient to explain the complex progression of MASLD/MASH. A more informative approach is to evaluate the integrated relationship among bile acid pool structure, metabolic compartment, and receptor expression.

### 2.3 Compartmental differences and heterogeneity in human studies

Human studies partly support the above interpretation, but they also demonstrate marked heterogeneity in the bile acid axis. In some MASLD populations, serum bile acid profiles are associated with disease severity. Certain secondary unconjugated bile acids and lithocholic acid-related molecules may be elevated in individuals with progressive fibrosis (30). However, in some non-cirrhotic MASH cohorts, plasma bile acids, FGF19, or 7α-hydroxy-4-cholesten-3-one (C4), a marker of bile acid synthesis, are not consistently associated with fibrosis stage after adjustment for body mass index (BMI), age, and metabolic comorbidities. Recent studies of plasma and fecal bile acids in advanced fibrotic MASLD also suggest that total plasma bile acids, primary/secondary bile acids, and chenodeoxycholic acid (CDCA)-family bile acids may increase, whereas enteric hormones or feedback markers do not necessarily change in parallel. These findings are more consistent with compartmental mismatch than with simple linear enhancement of a feedback axis (31).

Accordingly, neither the statement that “FXR activation is always beneficial” nor the statement that “increased bile acids are always pathogenic” adequately captures the current evidence. The direction, strength, and pathological meaning of bile acid signaling are jointly determined by the specific bile acid molecule, conjugation status, sampling compartment, and disease stage (32). Plasma bile acids more closely reflect spillover signals in the systemic circulation. Fecal bile acids mainly reflect microbial transformation and excretion in the intestinal lumen. Portal bile acids are more representative of gut-derived input, but they are difficult to obtain in human studies. Liver tissue bile acids and receptor expression are closer to local cellular responses. Only by integrating these compartmental differences with disease stage, metabolic comorbidities, and microbial function can the inconsistent results for bile acid-related markers in clinical MASLD studies be explained (17, 31).

## 3 Central interface: the microbiota–bile acid–intestinal barrier axis in gut–liver inflammatory crosstalk

### 3.1 Microbial functional enzymes remodel the bile acid pool: from taxonomic abundance to metabolic function

Within this bile acid-centered framework, the microbiota–bile acid–intestinal barrier interaction represents the central interface that best reflects the heterogeneity of MASLD/MASH. After primary bile acids synthesized in the liver enter the intestinal lumen, they do not simply undergo reabsorption or excretion. They are further processed by the gut microbiota (13, 14). Bile salt hydrolase (BSH) mediates the deconjugation of conjugated bile acids, 7α-dehydroxylation promotes the generation of secondary bile acids, and hydroxysteroid dehydrogenases and epimerization-related enzymes further alter the spatial configuration and receptor affinity of bile acid molecules (20, 22, 33). Through these microbial transformations, the functional properties of the bile acid pool are reshaped. Different bile acid molecules vary in hydrophobicity, membrane-perturbing capacity, intensity of FXR/TGR5 activation, immunomodulatory effects, and impact on the intestinal barrier (34).

At the taxonomic level, BSH activity is broadly distributed across several intestinal bacterial groups rather than being restricted to a single species. Representative BSH-positive taxa include Lactobacillus/Ligilactobacillus species, Bifidobacterium species, Enterococcus faecalis, Clostridium perfringens, and members of Bacteroides. Structurally and functionally characterized examples include BSH enzymes from Bifidobacterium longum, Enterococcus faecalis, Lactobacillus salivarius, and Clostridium perfringens (15, 33). In contrast, 7α-dehydroxylation is performed by a much narrower group of anaerobic bacteria carrying the bile acid-inducible bai gene cluster, with Clostridium scindens and related Clostridium species being the best-characterized examples (35, 36). Therefore, microbial bile acid metabolism in MASLD/MASH should be interpreted at both the taxonomic and functional levels: BSH-mediated deconjugation is relatively widespread, whereas secondary bile acid generation through 7α-dehydroxylation depends on more specialized microbial lineages.

The gut microbiota is therefore not merely a background component of bile acid metabolism. It functions as a metabolic “reprogrammer” that determines the direction and intensity of bile acid signaling. Microbial changes in MASLD/MASH should not be described only as shifts in taxonomic abundance. They are better understood as rearrangements of bile acid-metabolic functional modules (18). Compared with an increase or decrease in a specific genus, BSH activity, 7α-dehydroxylation capacity, secondary bile acid-generating networks, and bile acid metabolic flux are more reproducible, verifiable, and translatable mechanistic indicators (37). Human evidence has begun to support the clinical relevance of BSH activity in MASLD, although the evidence remains early and should not be interpreted as definitive causality. A 2026 Gut Microbes study reported that fecal BSH activity was elevated in patients with MASLD and advanced liver fibrosis, and that this activity correlated with reduced fecal bile acid levels. The same study further identified diverse BSH-active bacteria from patient fecal samples and showed that small-molecule BSH inhibitors could suppress BSH activity in fecal communities and monocultures without broadly impairing bacterial viability (38). These findings suggest that BSH activity may represent a measurable microbial function associated with MASLD progression, rather than only a taxonomic feature of the gut microbiota. However, the available human data are still limited, and it remains uncertain whether increased BSH activity is a driver, a disease-associated adaptation, or a marker of altered intestinal ecology during advanced fibrotic MASLD.

This concept has direct implications for study design. 16S ribosomal RNA (16S rRNA) sequencing can describe the taxonomic structure of the microbiota, but it cannot fully explain bile acid metabolic function. Different microbial communities may generate similar bile acid profiles through functional redundancy. Conversely, similar genus-level abundance may produce different BSH activities and secondary bile acid-generating capacities because of differences in gene expression, substrate availability, or ecological interactions (38). Future studies of the bile acid axis should therefore integrate metagenomics, metatranscriptomics, metabolomics, and enzyme activity assays. When feasible, metabolic flux analysis or isotope tracing should also be included, rather than inferring mechanism solely from changes in genus-level abundance (18).

### 3.2 Secondary bile acids alter receptor signaling properties: molecular context matters

In MASLD/MASH, microbiota-mediated bile acid reprogramming should be interpreted as a context-dependent interface rather than as a uniform pathogenic sequence. Dysbiosis, enrichment of hydrophobic secondary bile acids, intestinal barrier injury, and microbial inflammation-related input may occur together, but their direction and contribution vary across compartments and disease stages. Hydrophobic secondary bile acids, such as deoxycholic acid (DCA) and lithocholic acid (LCA), are representative molecules of particular interest. They may alter intestinal and hepatic FXR/TGR5 signaling outputs and thereby influence feedback regulation of bile acid synthesis, lipid metabolism, and immune regulation. Under barrier-disruptive or lipotoxic conditions, their membrane-perturbing and stress-promoting properties may also aggravate epithelial injury, weaken tight junction stability, and contribute to barrier dysfunction (39).

Secondary bile acids are not a homogeneous molecular group and should not be described as uniformly pathogenic. Animal model studies suggest that changes in the secondary bile acid pool in MASH are not necessarily explained entirely by gut microbial composition or microbial enzyme activity. Intrahepatic bile acid rehydroxylation may also participate in secondary bile acid profile remodeling (40). In addition, the LCA derivatives 3-oxoLCA and isoalloLCA can regulate T helper 17/regulatory T cell (Th17/Treg) differentiation, and microbial bile acid metabolites can help maintain intestinal RORγ+ Treg homeostasis (41, 42). Together, these findings indicate that the pathological significance of secondary bile acids depends on the specific molecule, spatial configuration, site of action, and local immune ecology, rather than on secondary bile acid status alone.

This evidence further indicates that the pathological meaning of a bile acid molecule depends on its source, configuration, compartment, and context of action. Protective bile acid derivatives present locally in the intestinal lumen may act indirectly by shaping microbial ecology. In contrast, hydrophobic bile acids entering the portal vein or liver may amplify inflammatory injury when barrier dysfunction and lipotoxicity are present. Bile acid research therefore needs to answer several questions simultaneously: which molecule is involved, in which compartment it acts, whether it operates through receptor-dependent or receptor-independent mechanisms, and which cell type or microbial niche is affected (43).

### 3.3 The intestinal barrier as the interface linking bile acid dysregulation to liver injury

Intestinal barrier disruption represents one of the main interfaces through which bile acid-axis dysregulation may become linked to liver injury. When the barrier is intact, bile acids mainly function as metabolic and signaling molecules within enterohepatic circulation. Once barrier integrity is impaired, abnormal bile acid profiles may be delivered to the portal circulation together with LPS, bacterial DNA, fungal components, and other microbial-associated molecules (44). In this setting, bile acid dysregulation is better viewed as a contributor to gut-derived inflammatory input rather than as an isolated metabolic abnormality. MASLD-associated dysbiosis can reduce the supply of barrier-protective factors, such as short-chain fatty acids and indole derivatives (45, 46). Dietary factors, including high fructose and high saturated fat intake, can also disrupt tight junction structures and increase intestinal permeability (47). After barrier function declines, microbial-associated molecular patterns can more readily enter the liver through the portal vein. At the same time, the portal input profile of bile acid metabolites may change, allowing unconjugated hydrophobic bile acids to act together with microbial products in the liver and further influence intrahepatic inflammation and the fibrotic microenvironment (38).

After entering the liver, LPS can activate Kupffer cells and monocyte-derived macrophages through toll-like receptor 4 (TLR4) and downstream inflammatory pathways, including myeloid differentiation primary response 88 (MyD88), nuclear factor κB (NF-κB), and the NLR family pyrin domain containing 3 (NLRP3) inflammasome. This process shifts a lesion initially dominated by metabolic stress toward inflammation, hepatocellular injury, and fibrosis. In this context, bile acids do not replace LPS or other microbial products as independent stimuli. Instead, they modify the stress thresholds of hepatocytes, immune cells, and cholangiocytes, making gut-derived inflammatory input more likely to be translated into intrahepatic inflammatory amplification (48).

The microbiota–bile acid–intestinal barrier axis should therefore be presented not as a general statement that the gut microbiota participates in MASLD, but as a specific interpretive interface linking microbial bile acid remodeling, barrier defects, and portal inflammatory input. Its key components include rearranged microbial metabolic function, impaired barrier integrity, functional remodeling of the bile acid pool, and the capacity of these changes to reach the liver and influence immune and mesenchymal cell responses (44, 49). From this perspective, the gut microbiota and its metabolites are not passive background factors in MASLD/MASH progression. They may represent an important regulatory interface in gut–liver inflammatory crosstalk and disease progression (19, 50).

### 3.4 Intestinal inflammation may reciprocally disrupt enterohepatic bile acid circulation

Intestinal inflammation should not be interpreted only as a terminal consequence of the microbiota–bile acid axis. Current evidence suggests that it may also act as an amplifying component that disrupts enterohepatic bile acid circulation. The ileum is a key site for bile acid reabsorption and FXR–FGF19 feedback. Its inflammatory status can alter ASBT-mediated bile acid reabsorption, ileal FXR signaling, and intrahepatic feedback regulation of bile acid synthesis (51, 52). A 2024 Journal of Hepatology study showed that MASLD-associated ileitis can shift ileal contents toward a microenvironment enriched in secondary bile acids. These secondary bile acids activate ileal cluster of differentiation 8-positive (CD8+) T cells through the TGR5/mechanistic target of rapamycin/oxidative phosphorylation (TGR5/mTOR/OXPHOS) pathway, aggravate ileal tissue injury, and further disturb enterohepatic bile acid circulation. This process can suppress hepatic FXR activation and thereby worsen the MASLD phenotype (53).

This finding reframes the intestine not as a passive recipient of hepatic bile acids, but as a regulatory interface that can reshape enterohepatic bile acid circulation in reverse. In this framework, the bile acid axis in MASLD/MASH is better understood as a reciprocal loop involving intrahepatic metabolic mismatch, intestinal bile acid remodeling, ileal inflammation, and impaired hepatic FXR feedback, rather than as a one-way pathway from the liver to the intestine. This loop also helps explain why plasma bile acids, fecal bile acids, FGF19, C4, and the proportion of secondary bile acids often show inconsistent results across studies. These markers do not measure the same event; they reflect different biological layers, including systemic spillover, intestinal transformation, intrahepatic synthetic feedback, and enterohepatic circulation efficiency (31, 54).

The significance of the microbiota–bile acid–intestinal barrier axis therefore lies not simply in showing that intestinal factors participate in MASLD/MASH, but in providing a framework to explain how remodeling of the luminal bile acid pool may be associated with portal inflammatory input. BSH activity, secondary bile acid-generating capacity, and changes in bile acid hydrophobicity can reflect microbiota-driven functional remodeling of the bile acid pool. These features are important observational nodes for subsequent mechanistic stratification (38). The supply of short-chain fatty acids and indole metabolites, together with mucus-layer integrity and tight junction status, affects intestinal barrier integrity and determines whether luminal metabolic events can be converted into gut-derived inflammatory input (44, 55). Downstream pathology ultimately depends on how these gut-derived inputs act on the inflammatory–fibrotic microenvironment composed of hepatocytes, macrophages, HSCs, and liver sinusoidal endothelial cells (43).

## 4 Downstream effects: bile acid signaling within the intrahepatic inflammatory–fibrotic niche

After upstream intrahepatic bile acid metabolic mismatch and intermediate microbiota–bile acid–intestinal barrier interactions have developed, the central downstream question is not whether bile acids independently induce fibrosis. Rather, it is how abnormal bile acid profiles act together with lipotoxicity, oxidative stress, and gut-derived inflammatory input on the intrahepatic cellular network. The bile acid axis enters the inflammatory–fibrotic niche mainly through three routes. First, hydrophobic or abnormally conjugated bile acids may aggravate hepatocellular stress and promote the release of DAMPs, chemokines, and extracellular vesicles (7, 56). Second, bile acid metabolites may act together with microbial-associated molecules, such as LPS, to lower the threshold for Kupffer cell and monocyte-derived macrophage inflammatory activation (57–59). Third, proinflammatory and profibrotic mediators released by macrophages can further drive HSC activation, myofibroblast transition, and extracellular matrix deposition (25, 60, 61). This section therefore focuses on how the bile acid axis is translated from metabolic imbalance into cellular niche remodeling, rather than reducing it to a linear effect of a single bile acid molecule on fibrosis.

### 4.1 Hepatocellular injury: convergence between bile acid stress and lipotoxicity

The downstream pathological significance of the bile acid axis largely depends on how intermediate gut–liver inputs act on intrahepatic cellular networks. As microbial function changes, hydrophobic secondary bile acids accumulate, and the intestinal barrier is disrupted, unconjugated bile acids, LPS, and other microbial metabolites can continuously enter the liver through the portal vein and overlap with intrahepatic lipotoxicity and oxidative stress. At this stage, hepatocytes are no longer passive carriers of lipid deposition. They progressively become cells that release injury signals. Bile acid stress, free cholesterol, saturated fatty acids, diacylglycerols (DAG), and ceramides jointly induce mitochondrial dysfunction, endoplasmic reticulum stress, impaired autophagy, and cell death, thereby promoting the release of DAMPs, chemokines, and extracellular vesicles (9, 25).

The role of bile acid receptors at this stage is strongly context dependent. TGR5 is a typical example. Conventional views often emphasize that TGR5 promotes glucagon-like peptide-1 (GLP-1) release, improves energy metabolism, and exerts anti-inflammatory effects in some immune cells. Recent studies, however, suggest that TGR5 is not invariably protective. A 2025 Advanced Science study showed that, in a lipotoxic environment, conjugated lithocholic acid can influence CD36-, TRIM21-, and BBOX1-related molecular events through hepatocyte TGR5. This suppresses *de novo* carnitine synthesis, weakens fatty acid oxidation, and aggravates lipotoxicity-related cell death and inflammation, thereby promoting the transition from MASLD to MASH (56). This finding indicates that the same bile acid receptor may produce different, or even opposite, biological outcomes depending on the cellular and ligand context.

Hepatocellular injury is not the terminal consequence of bile acid stress; it is also an initiating signal for downstream immune and mesenchymal cell remodeling (62). When bile acid stress overlaps with lipotoxicity, hepatocytes can release DAMPs, C-C motif chemokine ligand 2 (CCL2), C-X-C motif chemokine ligand 10 (CXCL10), extracellular vesicles, and other signals, thereby inducing Kupffer cell activation and monocyte recruitment (57). These inflammatory and chemotactic signals provide a sustained background for HSC activation. The effect of the bile acid axis on fibrosis is therefore better understood as a continuous process of hepatocellular injury signal amplification, immune cell recruitment, and mesenchymal cell activation, rather than as a single step in which bile acids directly act on HSCs (58).

### 4.2 Macrophage activation: intrahepatic amplification of portal inflammatory input

Abnormal bile acid profiles and microbial products entering through the portal vein form a key bridge between intermediate intestinal barrier disruption and downstream intrahepatic inflammation and fibrosis (16). LPS can activate Kupffer cells and monocyte-derived macrophages through the TLR4–MyD88–NF-κB pathway. Hydrophobic bile acids and their receptor signals may further alter the stress thresholds of hepatocytes and immune cells, making metabolic injury more likely to be translated into an amplified inflammatory response (58, 59). Once lipotoxicity, oxidative stress, and bile acid stress exceed the compensatory threshold of hepatocytes, injured hepatocytes release DAMPs, chemokines, and extracellular vesicles, which promote the recruitment, expansion, and phenotypic conversion of Kupffer cells and monocyte-derived macrophages (57, 62).

In this process, bile acid signaling is more than an additional stimulus alongside LPS. It may amplify the intrahepatic effects of portal inflammatory input by changing the extent of hepatocellular injury, chemokine release, and immune receptor activation (11). Particularly when the intestinal barrier is impaired, hydrophobic bile acids, conjugated bile acids, and LPS can enter the liver together. This co-exposure makes Kupffer cells and monocyte-derived macrophages more likely to maintain a proinflammatory state and promotes the release of tumor necrosis factor-α (TNF-α), interleukin-1β (IL-1β), interleukin-6 (IL-6), CCL2, and related mediators (58). The link between the bile acid axis and macrophages should therefore be understood as inflammatory threshold regulation and amplification of a profibrotic microenvironment, rather than as direct linear stimulation of macrophages by bile acids.

Macrophages are not a uniform proinflammatory cell population. They are highly plastic intrahepatic immune regulatory nodes (57, 62). Across different disease stages, tissue-resident Kupffer cells, C-C chemokine receptor 2 (CCR2)-related monocyte-derived macrophages, lipid-associated macrophages, and scar-associated macrophages may participate in inflammatory initiation, phagocytic clearance, fibrosis progression, or fibrosis regression. Lipid-associated macrophages generally refer to macrophage subsets enriched in lipid-rich or steatotic niches and characterized by lipid-handling and tissue-remodeling programs, whereas scar-associated macrophages refer to macrophage populations located within or adjacent to fibrotic scar areas that participate in extracellular matrix remodeling and fibrosis regression or progression depending on disease context (57, 62–64). Bile acid signaling may indirectly shape the functional direction of these macrophage subsets by altering hepatocellular injury, chemokine release, gut-derived inflammatory input, and immune receptor activation (11, 58). Therefore, downstream studies of the bile acid axis should not focus only on receptor expression in hepatocytes. Immune cell states should also be incorporated into the same explanatory framework.

### 4.3 HSC activation and the fibrotic niche: from injury suppression to restoration of the repair niche

HSC activation is a downstream outcome of the pathological effects of the bile acid axis. This does not imply that bile acids necessarily act directly on HSCs. A more cautious interpretation is that abnormal bile acid profiles indirectly provide persistent stimulation for HSC activation by aggravating hepatocellular stress, enhancing portal inflammatory input, and promoting profibrotic macrophage responses (25, 62).

Persistent inflammatory input can ultimately drive HSC activation and extracellular matrix deposition. Macrophages secrete transforming growth factor-β (TGF-β), platelet-derived growth factor (PDGF), IL-1β, TNF-α, CCL2, and other mediators, which promote the transition of HSCs from a quiescent state to a myofibroblast-like phenotype and enhance collagen deposition and extracellular matrix remodeling (58). Liver sinusoidal endothelial cells, ductular reaction-related cells, platelet-derived factors, and extracellular vesicles may also participate in this process. The resulting process is not a one-way linear pathway, but a fibrosis-promoting niche maintained by continuous communication among injured hepatocytes, macrophages, HSCs, and endothelial cells (65, 66).

Recent single-cell and spatial omics studies further indicate that MASH fibrosis is not an isolated response of a single cell population. It is a tissue microenvironment jointly shaped by hepatocellular stress, cholangiocyte/epithelial-like cell plasticity, accumulation of scar-associated macrophages, mesenchymal cell activation, and intercellular paracrine signaling (65, 67). A 2026 Cell Reports study in a mouse model of MASH fibrosis regression found Wnt9b–Sfrp2-related crosstalk between scar-associated endothelial cells and HSCs. Disrupting this signal weakened spontaneous fibrosis regression (68). This finding does not mean that bile acids directly drive fibrosis reversal. Rather, it suggests that MASH fibrosis research needs to move beyond blocking injury signals toward restoration of the tissue repair niche (68).

The downstream significance of the bile acid axis is therefore not to identify a single pathogenic bile acid or a single protective receptor. Its value lies in clarifying which signaling nodes may determine the transition from metabolic stress to inflammatory–fibrotic remodeling across disease stages, cellular compartments, and bile acid profile backgrounds. In early disease, bile acid signaling is mainly embedded in intrahepatic metabolic reprogramming. Once the inflammatory stage is reached, abnormal bile acids, toxic lipids, gut-derived LPS, and oxidative stress may act together to amplify hepatocellular injury. In the fibrotic stage, bile acid signaling is more deeply embedded in the pathological niche composed of hepatocytes, macrophages, endothelial cells, and HSCs. It may influence intercellular communication, extracellular matrix deposition, and the potential for fibrosis regression (25, 69). The major mechanistic nodes discussed above, together with their supporting evidence and major limitations, are summarized in Table 1.

**Table 1:** Evidence map of bile acid-axis mechanisms in MASLD/MASH.

Mechanistic module	Evidence base	Support	Interpretation	Limitation
Altered bile acid pool composition	Human metabolomics, cohort studies, systematic reviews	Moderate	Reflects altered pool composition, conjugation status, hydrophobicity, compartmental distribution, and receptor-mediated signaling. It should not be interpreted only as a change in total bile acid levels.	Human findings remain heterogeneous across plasma, fecal, liver tissue, and disease stages.
Impaired FXR–FGF19 feedback and hepatic bile acid homeostasis	Biomarker studies, receptor biology, transporter studies, translational studies	Moderate	Disturbed ileal FXR–FGF19 signaling, CYP7A1/CYP8B1-regulated synthesis, and transporter imbalance may jointly reshape intrahepatic and luminal bile acid exposure.	FGF19, C4, and transporter-related findings are inconsistent across cohorts, and direct human causal evidence remains incomplete.
Microbial bile acid transformation	Metagenomics, bile acid profiling, microbial enzyme assays, animal models	Moderate to strong	BSH, 7α-dehydroxylation enzymes, HSDHs, and epimerization-related enzymes remodel the bile acid pool by changing conjugation, secondary bile acid generation, hydrophobicity, receptor affinity, and immune-regulatory properties.	Functional redundancy among microbial taxa complicates causal attribution; disease-specific human validation of individual enzyme modules remains limited.
Intestinal barrier dysfunction and portal inflammatory input	Gut permeability studies, barrier markers, animal models, translational human evidence	Strong	Barrier impairment permits luminal bile acids, LPS, microbial DNA, fungal components, and other microbial products to enter the portal circulation and amplify hepatic inflammatory input.	Human barrier assessment lacks standardized, compartment-specific methods, and portal sampling is rarely available.
Ileal inflammation and enterohepatic feedback disruption	Translational studies, animal models, bile acid and immune profiling	Moderate	Ileal inflammation can alter bile acid reabsorption, weaken ileal FXR–FGF19 feedback, disturb hepatic bile acid synthesis, and form a self-amplifying gut–liver loop.	It remains difficult to distinguish cause, consequence, and compensatory adaptation in human MASLD/MASH.
Hepatocellular bile acid stress and lipotoxicity	Metabolic injury models, receptor studies, human tissue evidence	Moderate to strong	Bile acid stress may converge with lipotoxicity, oxidative stress, ER stress, mitochondrial dysfunction, and impaired autophagy, promoting DAMPs, chemokines, extracellular vesicles, and hepatocellular injury.	The contribution of individual bile acid species is difficult to separate from broader metabolic injury.
Macrophage activation and inflammatory amplification	Single-cell studies, spatial omics, macrophage biology, mechanistic models	Strong	Bile acid-associated stress and gut-derived inflammatory input can indirectly shape Kupffer cell and monocyte-derived macrophage activation, amplifying inflammatory and profibrotic signaling.	Direct bile acid-specific effects on macrophage subsets remain context dependent and incompletely defined.
HSC activation and fibrotic niche remodeling	Fibrosis biology, single-cell/spatial omics, mechanistic studies	Strong	Bile acid-axis abnormalities converge on HSC activation mainly through hepatocellular injury, macrophage activation, endothelial–mesenchymal crosstalk, and inflammatory niche remodeling rather than through a single direct profibrotic pathway.	Direct evidence that specific bile acids independently drive HSC activation in human MASH remains limited.
Context-dependent FXR/TGR5 signaling and stratification potential	Receptor biology, therapeutic trials, biomarker studies, translational research	Preliminary to moderate	FXR/TGR5 effects vary by ligand, cell type, compartment, receptor expression, and disease stage. Multi-compartment bile acid profiling, microbial enzyme activity, barrier status, and tissue-specific receptor signaling may support stratification and response monitoring.	Overgeneralization from single-receptor or single-ligand models remains a major risk; biomarker standardization and prospective validation are still insufficient.

Strong support indicates convergent evidence from human studies and mechanistic experimental data. Moderate support indicates biologically plausible evidence supported by several studies but limited by cohort heterogeneity, compartmental inconsistency, or incomplete causal validation. Preliminary support indicates emerging or exploratory evidence requiring further confirmation. BSH, bile salt hydrolase; C4, 7α-hydroxy-4-cholesten-3-one; DAMPs, damage-associated molecular patterns; FXR, farnesoid X receptor; FGF19, fibroblast growth factor 19; HSC, hepatic stellate cell; HSDH, hydroxysteroid dehydrogenase; LPS, lipopolysaccharide; MASH, metabolic dysfunction-associated steatohepatitis; MASLD, metabolic dysfunction-associated steatotic liver disease; TGR5, G protein-coupled bile acid receptor 1.

## 5 Therapeutic translation: from bile acid receptor targeting to mechanism-based stratification and combination therapy

The bile acid axis entered MASH drug development relatively early among metabolic pathways (70). However, its translational value should not be reduced to pharmacological activation or inhibition of a single receptor. More importantly, it provides a framework for linking feedback regulation of bile acid synthesis, enterohepatic circulation, lipid metabolism, barrier function, and inflammatory–fibrotic responses. Existing clinical studies suggest that bile acid signaling can influence MASH-related histological endpoints, but strong intervention on a single receptor is unlikely to address the full pathological background of MASH.

FXR agonists represented by obeticholic acid (OCA) (71) have provided important validation for bile acid receptor-targeted therapy. The REGENERATE study (72) showed that OCA 25 mg increased the response rate for fibrosis improvement by at least one stage without worsening of NASH, suggesting that FXR modulation has certain antifibrotic potential. At the same time, OCA development also exposed the limitations of systemic FXR activation (71), including pruritus, increased low-density lipoprotein cholesterol (LDL-C), cholelithiasis, and potential hepatobiliary safety concerns. During regulatory review, the US FDA also emphasized that uncertainty remains regarding the relationship between histological surrogate endpoint benefits and long-term clinical benefits, and that long-term safety requires careful evaluation. Thus, FXR is a biologically plausible target, but it should not be equated with a universal therapeutic pathway suitable for simple, sustained, and strong systemic activation.

FGF19 analogs (73, 74) represent another strategy that mimics intestinal feedback. The rationale is to enhance intestinal FXR–FGF19 feedback, suppress intrahepatic bile acid synthesis, and improve hepatic lipid metabolism and metabolic stress. In some studies, drugs such as aldafermin (73, 74) showed potential to reduce liver fat content, improve aminotransferases, and improve some non-invasive fibrosis-related indicators. However, evidence remains insufficient regarding histological fibrosis improvement, long-term clinical outcomes, and identification of the most responsive populations. Therefore, the FGF19 pathway is currently more appropriately viewed as a candidate module for mechanistic stratification and combination strategies. It should not be described as a mature stand-alone therapeutic strategy.

Notably, the clearest clinical breakthroughs in the current MASH treatment field have not come from bile acid-axis drugs, but from broader metabolic therapies. This therapeutic pattern helps explain why bile acid-directed therapy has not become the dominant treatment strategy despite strong mechanistic rationale. MASH progression is driven by overlapping metabolic stress, lipotoxicity, inflammation, immune remodeling, and fibrosis; therefore, modulation of a single bile acid receptor or feedback pathway may be insufficient for many patients. In addition, FXR agonism has been constrained by pruritus, LDL-C elevation, gallbladder-related events, hepatobiliary safety concerns, and uncertain long-term benefit, whereas FGF19-related approaches still require clearer evidence for histological fibrosis improvement, long-term safety, and responsive patient selection (72, 73). By contrast, therapies targeting broader metabolic drivers, including THR-β agonism, GLP-1 receptor agonism, dual incretin agonism, glucagon/GLP-1 dual agonism, and FGF21-related pathways, have shown clinically meaningful effects on steatohepatitis resolution, fibrosis improvement, body weight, liver fat, or metabolic risk factors in recent trials (75–78). These findings suggest that the bile acid axis may be most useful as a stratification, monitoring, and combination-therapy module rather than as a stand-alone dominant therapeutic pathway.

The thyroid hormone receptor-β (THR-β) agonist resmetirom (75) has received accelerated approval from the US FDA for adults with non-cirrhotic MASH and moderate-to-advanced fibrosis. The MAESTRO-NASH study (75) showed that resmetirom was superior to placebo for both histological endpoints: MASH/NASH resolution without worsening of fibrosis and fibrosis improvement without worsening of disease activity. The glucagon-like peptide-1 (GLP-1) receptor agonist semaglutide (79, 80) also showed signals of MASH resolution and fibrosis improvement in the phase III ESSENCE study (76). In 2025, it received accelerated approval from the US FDA for adults with non-cirrhotic MASH and moderate-to-advanced fibrosis. Resmetirom and semaglutide are not bile acid-axis drugs and should not be used as direct evidence for bile acid-targeted therapy. Nevertheless, they indicate that MASH treatment is shifting from single-pathway targeting toward integrated intervention addressing metabolic burden, inflammatory activity, and fibrosis risk.

Based on this shift, the bile acid axis may be more appropriately positioned as a mechanistic module for stratification, monitoring, and combination-therapy design, rather than as a stand-alone pathway capable of addressing the full therapeutic spectrum of MASH. For patients dominated by impaired intrahepatic feedback regulation of bile acid synthesis, FXR–FGF19-related indicators may have stratification value. For patients dominated by abnormal microbial bile acid transformation and intestinal barrier disruption, BSH activity, secondary bile acid-generating capacity, bile acid hydrophobicity, barrier integrity, and portal inflammatory input may better reflect disease-driving factors. In patients who have already entered the inflammatory–fibrotic stage, bile acid signaling should be assessed together with hepatocellular injury, macrophage activation, HSC activation, and the fibrotic niche.

Microbiota-directed interventions further illustrate this translational potential, but they should be interpreted cautiously. Antibiotics can rapidly remodel bile acid metabolism by depleting bile acid-transforming bacteria and altering secondary bile acid generation, FXR signaling, and downstream metabolic responses; however, these effects are highly context dependent and may be either protective or harmful depending on the diet, microbial community, host disease stage, and antibiotic spectrum (81–83). In contrast, probiotics, prebiotics, and synbiotics may improve MASLD-related metabolic or inflammatory indices by reshaping microbial composition, strengthening the intestinal barrier, increasing short-chain fatty acid and indole-related metabolites, and partially normalizing bile acid profiles (84–87). Nevertheless, current human evidence remains heterogeneous, and most studies rely on biochemical, microbiome, or non-invasive fibrosis markers rather than histological fibrosis endpoints. Therefore, antibiotics and probiotics should not be presented as established antifibrotic therapies for MASLD/MASH, but rather as microbiota-directed tools that may help interrogate or modulate bile acid-related gut–liver mechanisms.

The translational value of the bile acid axis should therefore not be summarized as developing a bile acid drug. A more prudent direction is to establish a stratification framework based on bile acid profiles, microbial functional enzymes, intestinal barrier status, and tissue-specific receptor signaling. This framework can then be combined with weight loss therapy, THR-β agonists, GLP-1 receptor agonists, and potential anti-inflammatory or antifibrotic strategies. Recent studies of tirzepatide (88), survodutide (77), lanifibranor (89) and pegozafermin (78) also suggest that MASH treatment is moving toward a multi-pathway, stratified, and combination-therapy framework. This positioning preserves the mechanistic integrative value of the bile acid axis while avoiding oversimplification of FXR, TGR5, FGF19, or microbial functional modules as single therapeutic targets. The therapeutic implications of bile acid-axis modulation, including receptor targeting, microbiota-directed approaches, barrier-focused strategies, and mechanism-based stratification, are summarized in Table 2.

**Table 2:** Therapeutic implications of bile acid-axis modulation in MASLD/MASH.

Target/strategy	Approach	Evidence stage	Positioning	Caution
FXR agonism	Obeticholic acid and other FXR agonists	Phase III and regulatory experience	Validates FXR as a biologically relevant target and suggests antifibrotic potential in selected patients.	Limited by pruritus, LDL-C increase, gallbladder-related events, hepatobiliary safety concerns, and uncertain long-term clinical benefit.
FXR–FGF19 feedback enhancement	Aldafermin and other FGF19 analogs	Phase II and translational studies	May suppress hepatic bile acid synthesis, reduce liver fat, and improve aminotransferases or non-invasive metabolic stress markers.	Histological fibrosis benefit, long-term safety, and responsive populations remain insufficiently defined.
TGR5-related modulation	Experimental or tissue-selective TGR5 modulation	Preclinical to early translational research	May influence GLP-1 release, energy metabolism, immune tone, and inflammatory thresholds.	Highly context dependent; effects may differ across hepatocytes, immune cells, ileal CD8+ T cells, and enteroendocrine cells.
Microbiota-directed bile acid remodeling	Probiotics, prebiotics, engineered bacteria, BSH modulation, or other microbiota-targeted approaches	Preclinical, translational, and early human exploratory evidence	May reshape luminal bile acid composition, reduce harmful bile acid profiles, and attenuate gut–liver inflammatory crosstalk.	Human efficacy is not established; microbial functional redundancy and ecological complexity limit predictability.
Intestinal barrier-focused strategies	Dietary intervention, fiber-related approaches, gut barrier protection, or permeability reduction	Preclinical, translational, and supportive clinical rationale	May reduce portal inflammatory input and weaken gut-derived amplification of hepatic inflammation.	Barrier biomarkers, intervention endpoints, and patient selection criteria remain poorly standardized.
Combination with broader metabolic therapies	Weight loss, THR-β agonists, GLP-1 receptor agonists, dual/triple incretin agents, or FGF21-related agents with bile acid-axis stratification	Clinical and emerging translational framework	May address metabolic burden while using bile acid-axis features for patient stratification and treatment-response monitoring.	Combination efficacy, safety, treatment sequencing, and biomarker-guided selection require prospective validation.
Mechanism-based stratification and monitoring	Plasma/fecal bile acid profiling, FGF19, C4, BSH activity, barrier markers, and tissue-specific receptor signatures	Translational and biomarker-development stage	May identify bile acid-axis-dominant phenotypes and guide rational combination therapy.	Not a therapy itself; assay standardization and prospective clinical validation remain insufficient.

This table summarizes the translational relevance of bile acid-axis-related therapeutic strategies in MASLD/MASH. Current evidence supports positioning the bile acid axis primarily as a mechanism-based stratification, monitoring, and combination-therapy module rather than as a uniformly effective stand-alone treatment strategy. BSH, bile salt hydrolase; C4, 7α-hydroxy-4-cholesten-3-one; FXR, farnesoid X receptor; FGF19, fibroblast growth factor 19; GLP-1, glucagon-like peptide-1; LDL-C, low-density lipoprotein cholesterol; MASH, metabolic dysfunction-associated steatohepatitis; MASLD, metabolic dysfunction-associated steatotic liver disease; TGR5, G protein-coupled bile acid receptor 1; THR-β, thyroid hormone receptor-β.

## 6 Discussion: heterogeneity, evidence boundaries, and future directions for bile acid-axis research

The role of the bile acid axis in MASLD/MASH has received increasing attention, but current findings remain clearly heterogeneous. In this context, the contribution of this review should be distinguished from a claim of complete originality. Several recent reviews have summarized bile acid signaling, gut–liver interactions, and microbiota-derived metabolic pathways in MASLD/MASH (17–19). The specific contribution of the present review is to frame these scattered findings as a cautious three-layer model: upstream intrahepatic bile acid maladaptation, an intermediate microbiota–bile acid–intestinal barrier interface, and downstream inflammatory–fibrotic niche remodeling. This model is intended to clarify interpretation, compartmental differences, and evidence boundaries rather than to assert a new causal mechanism. Observations of total bile acids, primary/secondary bile acids, conjugated/unconjugated bile acids, FGF19, and C4 are not fully consistent across cohorts (31, 54). This heterogeneity should not be attributed simply to inadequate study quality, nor should it be used to deny the pathological significance of the bile acid axis. A more reasonable explanation is that bile acid signaling is influenced by sampling compartment, disease stage, and population heterogeneity.

Different sampling compartments do not reflect the same biological event. Plasma bile acids more often represent spillover signals in the systemic circulation and are easily influenced by the extent of liver injury, bile acid clearance capacity, and enterohepatic circulation efficiency. Fecal bile acids mainly reflect microbial transformation and excretion in the intestinal lumen. Portal bile acids are closer to gut-derived input but are difficult to obtain in human studies. Liver tissue bile acids and receptor expression more directly reflect local cellular responses (13). If sampling compartments are not distinguished, simple comparisons of increased or decreased bile acids can easily lead to conflicting conclusions.

Several areas of conflicting evidence deserve explicit consideration. First, FXR agonism supports the biological relevance of bile acid signaling, but clinical translation has been limited by pruritus, lipid changes, gallbladder-related events, hepatobiliary safety concerns, and uncertainty regarding long-term clinical benefit (72). Second, FGF19-related strategies may reduce liver fat and improve biochemical or non-invasive markers, but histological fibrosis benefit and the identification of responsive populations remain incompletely established (73). Third, circulating bile acid studies show substantial heterogeneity across disease severity, geographic background, and bile acid species; for example, meta-analytic evidence suggests altered circulating bile acids in MASLD, whereas a recent advanced-fibrosis cohort found discordant plasma and fecal bile acid profiles and no parallel increase in FGF19 or GLP-1 (31, 54). Fourth, animal studies do not always map directly onto human MASLD/MASH, as bile acid pool composition, microbial ecology, diet, and host metabolic status can differ substantially; one MASH mouse study even suggested that bile acid pool changes may occur through a gut microbiota-independent mechanism (40). These inconsistencies support a cautious interpretation in which bile acid-axis findings are stratified by receptor, compartment, disease stage, microbial function, and host metabolic context rather than treated as a uniform pathogenic pathway.

The effects of bile acid receptors are also strongly tissue and cell specific. FXR does not have identical functions in the liver and intestine. Hepatic FXR is more directly involved in bile acid synthesis, lipid metabolism, and inflammatory regulation, whereas intestinal FXR mainly influences intrahepatic bile acid production and metabolic homeostasis through FGF19 feedback (10). The role of TGR5 depends even more on ligand type and the local microenvironment. It may produce different effects in enteroendocrine cells, immune cells, hepatocytes, and inflammatory states in the ileum (56, 90). Thus, defining FXR or TGR5 simply as a protective receptor or an injury-promoting receptor does not reflect the current evidence. The more important question is which receptor signals have true pathological significance in a given disease stage, compartment, and bile acid profile background (32).

Existing studies have not fully resolved the boundary between association and causality. Therefore, the framework proposed in this review should be understood as an integrative and hypothesis-generating model rather than as proof of a single causal pathway. Its purpose is to organize heterogeneous evidence from human cohorts, experimental models, microbiota studies, and therapeutic observations, while acknowledging that the direction and strength of causality may differ across disease stages, sampling compartments, microbial configurations, and bile acid species. Many cohort studies have identified correlations among bile acid profiles, microbial composition, barrier markers, and fibrosis severity, but these data cannot directly prove that a specific bile acid molecule or microbial change drives disease progression. Animal experiments and *in vitro* studies help validate mechanisms, but their bile acid composition, microbial structure, dietary background, and metabolic status are not fully consistent with human MASLD/MASH (40, 43). Future research therefore needs to combine human cohorts, organoids, germ-free or colonized models, isotope tracing, multi-omics, and intervention studies to distinguish accompanying changes, compensatory changes, and pathological nodes with true causal relevance (38).

In addition, bile acid-axis research still faces translational boundaries. Studies of OCA and FGF19 analogs show that bile acid signaling can affect MASH-related endpoints, but they also suggest that strong intervention on a single pathway may be limited by safety concerns, therapeutic heterogeneity, and unclear identification of responsive populations (71, 73). With resmetirom and semaglutide entering clinical practice, the therapeutic landscape of MASH is moving from single-mechanism targeting toward integrated metabolic management and pathology-based stratified intervention (75, 76). In this context, the bile acid axis is better positioned as a mechanistic module for stratification, monitoring, and combination-therapy design, rather than as a central target replacing all therapeutic strategies (70).

Future studies should prioritize three areas. First, multi-compartment bile acid profiling should integrate plasma, fecal, portal, and liver tissue findings rather than using a single marker to represent the entire bile acid axis. Second, microbiota research should move beyond taxonomic abundance toward functional enzymes, metabolic flux, and barrier status, thereby clarifying which intermediate changes are associated with portal inflammatory input and intrahepatic injury (91). Third, single-cell and spatial omics should localize bile acid receptor signaling to specific cell populations and fibrotic niches, helping to identify disease-stage-specific nodes with intervention value (92). These priorities may help move the bile acid axis from a mechanistic hypothesis toward a verifiable, stratifiable, and translatable module.

## 7 Conclusions

The bile acid axis provides an integrative, but still evidence-bounded, mechanistic perspective for understanding MASLD/MASH progression. Current evidence suggests that bile acid dysregulation should not be summarized simply as increased or decreased total bile acids. Rather, it should be understood as a systemic mismatch among bile acid pool composition, conjugation status, microbial transformation, compartmental distribution, and receptor-mediated signaling output. This mismatch links bile acid signaling to intrahepatic metabolic reprogramming, abnormal enterohepatic circulation, and inflammatory–fibrotic responses, although the strength and direction of these links remain context dependent (13, 31).

Within this mechanistic continuum, microbiota–bile acid–intestinal barrier interactions appear to be an important interface connecting upstream metabolic abnormalities with downstream intrahepatic inflammation and fibrosis. The gut microbiota remodels the bile acid pool through functional enzymes, whereas intestinal barrier status determines whether luminal metabolic events can be converted into portal inflammatory input. Intrahepatic immune and mesenchymal cell responses then shape the final pathological consequences. Therefore, the core value of the bile acid axis does not lie in identifying a single pathogenic bile acid or a single protective receptor. Instead, it lies in clarifying the conditional coupling among intrahepatic metabolism, intestinal ecology, and the fibrotic microenvironment (14).

Future work should move the bile acid axis from descriptive association toward a verifiable, stratifiable, and translatable mechanistic module. This will require multi-compartment bile acid profiling, microbial functional-enzyme assessment, intestinal barrier evaluation, and tissue-specific receptor signaling analysis in the same study framework. In clinical translation, the bile acid axis is most appropriately positioned as a tool for mechanistic subtyping, treatment-response monitoring, and rational combination-therapy design, rather than as a stand-alone explanation for all MASLD/MASH progression (53, 93).

## References

[1] Rinella ME Neuschwander-Tetri BA Siddiqui MS Abdelmalek MF Caldwell S Barb D . AASLD Practice Guidance on the clinical assessment and management of nonalcoholic fatty liver disease. Hepatology. (2023) 77:1797–835. doi: 10.1097/HEP.0000000000000323 36727674 PMC10735173

[2] Riazi K Azhari H Charette JH Underwood FE King JA Afshar EE . The prevalence and incidence of NAFLD worldwide: a systematic review and meta-analysis. Lancet Gastroenterol Hepatol. (2022) 7:851–61. doi: 10.1016/S2468-1253(22)00165-0 35798021

[3] Lazarus JV Mark HE Anstee QM Arab JP Batterham RL Castera L . Advancing the global public health agenda for NAFLD: a consensus statement. Nat Rev Gastroenterol Hepatol. (2022) 19:60–78. doi: 10.1038/s41575-021-00523-4 34707258

[4] Rinella ME Lazarus JV Ratziu V Francque SM Sanyal AJ Kanwal F . A multisociety Delphi consensus statement on new fatty liver disease nomenclature. Hepatology. (2023) 78:1966–86. doi: 10.1097/HEP.0000000000000520 37363821 PMC10653297

[5] European European Association for the Study of the Liver European European Association for the Study of Diabetes European European Association for the Study of Obesity. EASL-EASD-EASO Clinical Practice Guidelines on the management of metabolic dysfunction-associated steatotic liver disease (MASLD). J Hepatol. (2024) 81:492–542. doi: 10.1016/j.jhep.2024.04.031 38851997

[6] Powell EE Wong VW Rinella M. Non-alcoholic fatty liver disease. Lancet. (2021) 397:2212–24. doi: 10.1016/S0140-6736(20)32511-3 33894145

[7] Loomba R Friedman SL Shulman GI. Mechanisms and disease consequences of nonalcoholic fatty liver disease. Cell. (2021) 184:2537–64. doi: 10.1016/j.cell.2021.04.015 33989548 PMC12168897

[8] Targher G Tilg H Byrne CD. Non-alcoholic fatty liver disease: a multisystem disease requiring a multidisciplinary and holistic approach. Lancet Gastroenterol Hepatol. (2021) 6:578–88. doi: 10.1016/S2468-1253(21)00020-0 33961787

[9] McGlinchey AJ Govaere O Geng D Ratziu V Allison M Bousier J . Metabolic signatures across the full spectrum of non-alcoholic fatty liver disease. JHEP Rep. (2022) 4:100477. doi: 10.1016/j.jhepr.2022.100477 35434590 PMC9006858

[10] Perino A Demagny H Velazquez-Villegas L Schoonjans K. Molecular physiology of bile acid signaling in health, disease, and aging. Physiol Rev. (2021) 101:683–731. doi: 10.1152/physrev.00049.2019 32790577

[11] Godlewska U Bulanda E Wypych TP. Bile acids in immunity: Bidirectional mediators between the host and the microbiota. Front Immunol. (2022) 13:949033. doi: 10.3389/fimmu.2022.949033 36052074 PMC9425027

[12] Shapiro H Kolodziejczyk AA Halstuch D Elinav E. Bile acids in glucose metabolism in health and disease. J Exp Med. (2018) 215:383–96. doi: 10.1084/jem.20171965 29339445 PMC5789421

[13] Collins SL Stine JG Bisanz JE Okafor CD Patterson AD. Bile acids and the gut microbiota: metabolic interactions and impacts on disease. Nat Rev Microbiol. (2023) 21:236–47. doi: 10.1038/s41579-022-00805-x 36253479 PMC12536349

[14] Guzior DV Quinn RA. Review: microbial transformations of human bile acids. Microbiome. (2021) 9:140. doi: 10.1186/s40168-021-01101-1 34127070 PMC8204491

[15] Foley MH O'Flaherty S Barrangou R Theriot CM. Bile salt hydrolases: Gatekeepers of bile acid metabolism and host-microbiome crosstalk in the gastrointestinal tract. PLoS Pathog. (2019) 15:e1007581. doi: 10.1371/journal.ppat.1007581 30845232 PMC6405046

[16] Agus A Clement K Sokol H. Gut microbiota-derived metabolites as central regulators in metabolic disorders. Gut. (2021) 70:1174–82. doi: 10.1136/gutjnl-2020-323071 33272977 PMC8108286

[17] Simbrunner B Paternostro R Reiberger T Trauner M. Bile acid signaling in MASLD: From pathogenesis to therapeutic applications. Hepatology. (2025). doi: 10.1097/HEP.0000000000001539. [Epub ahead of print]. 40971685

[18] Zhou J Zhu B Bing Z Wang T Zhao Y. The gut-liver axis in MASLD: from host-microbiome crosstalk to precision therapeutics. Microorganisms. (2026) 14:471. doi: 10.3390/microorganisms14020471 41753757 PMC12942669

[19] Cui C Gao S Shi J Wang K. Gut-Liver Axis: The role of intestinal microbiota and their metabolites in the progression of metabolic dysfunction-associated steatotic liver disease. Gut Liver. (2025) 19:479–507. doi: 10.5009/gnl240539 40336226 PMC12261135

[20] Wise JL Cummings BP. The 7-alpha-dehydroxylation pathway: an integral component of gut bacterial bile acid metabolism and potential therapeutic target. Front Microbiol. (2022) 13:1093420. doi: 10.3389/fmicb.2022.1093420 36699589 PMC9868651

[21] Chiang JYL Ferrell JM. Bile acid receptors FXR and TGR5 signaling in fatty liver diseases and therapy. Am J Physiol Gastrointest Liver Physiol. (2020) 318:G554–G73. doi: 10.1152/ajpgi.00223.2019 31984784 PMC7099488

[22] Foley MH O'Flaherty S Allen G Rivera AJ Stewart AK Barrangou R . Lactobacillus bile salt hydrolase substrate specificity governs bacterial fitness and host colonization. Proc Natl Acad Sci U S A. (2021) 118:e2017709118. doi: 10.1073/pnas.2017709118 33526676 PMC8017965

[23] Schreuder TC Marsman HA Lenicek M van Werven JR Nederveen AJ Jansen PL . The hepatic response to FGF19 is impaired in patients with nonalcoholic fatty liver disease and insulin resistance. Am J Physiol Gastrointest Liver Physiol. (2010) 298:G440–5. doi: 10.1152/ajpgi.00322.2009 20093562

[24] Kahl S Strassburger K Pacini G Trinks N Pafili K Mastrototaro L . Dysglycemia and liver lipid content determine the relationship of insulin resistance with hepatic OXPHOS capacity in obesity. J Hepatol. (2025) 82:417–26. doi: 10.1016/j.jhep.2024.08.012 39218222

[25] Kisseleva T Brenner D. Molecular and cellular mechanisms of liver fibrosis and its regression. Nat Rev Gastroenterol Hepatol. (2021) 18:151–66. doi: 10.1038/s41575-020-00372-7 33128017

[26] Ma J Ma Y Wan X Li J Zhang Y Liu J . Metabolic and genetic mechanisms of metabolic dysfunction-associated steatotic liver disease: an integrative perspective from molecular pathways to clinical challenges. Front Endocrinol. (2025) 16:1639064. doi: 10.3389/fendo.2025.1639064 41001677 PMC12457186

[27] Tang X Zhou Y Xia L Lin X Zhu Y Chen M . Multifaceted Interactions Between Bile Acids, Their Receptors, and MASH: From Molecular Mechanisms to Clinical Therapeutics. Molecules. (2025) 30:3066. doi: 10.3390/molecules30153066 40807240 PMC12348650

[28] Zhong S Chevre R Castano Mayan D Corliano M Cochran BJ Sem KP . Haploinsufficiency of CYP8B1 associates with increased insulin sensitivity in humans. J Clin Invest. (2022) 132:e152961. doi: 10.1172/JCI152961 36107630 PMC9621133

[29] Bilson J Scorletti E Swann JR Byrne CD. Bile Acids as Emerging Players at the Intersection of Steatotic Liver Disease and Cardiovascular Diseases. Biomolecules. (2024) 14:841. doi: 10.3390/biom14070841 39062555 PMC11275019

[30] Leng L Zhou G Liu A Wang H Ni Y. Lithocholic Acid Species: Metabolism, Signaling Pathways, and Clinical Significance in Enterohepatic Diseases. Int J Mol Sci. (2025) 26:11530. doi: 10.3390/ijms262311530 41373683 PMC12692668

[31] Pradeau M Beaulieu S Paquet V Trottier J Verreault M Ferland S . Plasma and fecal bile acids profiles in metabolic dysfunction-associated steatotic liver disease with advanced fibrosis. Am J Physiol Endocrinol Metab. (2025) 329:E644–E54. doi: 10.1152/ajpendo.00346.2024 40987517

[32] Peng Q Hao L Li S Yu F Li N Hu X . Critical review of natural products driven correction of bile acid dysregulation: a therapeutic strategy for nonalcoholic fatty liver disease. Front Pharmacol. (2025) 16:1640873. doi: 10.3389/fphar.2025.1640873 41357887 PMC12678328

[33] Dong Z Lee BH. Bile salt hydrolases: structure and function, substrate preference, and inhibitor development. Protein Sci. (2018) 27:1742–54. doi: 10.1002/pro.3484 30098054 PMC6199152

[34] Ma H Wang K Jiang C. Microbiota-derived bile acid metabolic enzymes and their impacts on host health. Cell Insight. (2025) 4:100265. doi: 10.1016/j.cellin.2025.100265 40814400 PMC12345280

[35] Vital M Rud T Rath S Pieper DH Schlüter D. Diversity of Bacteria Exhibiting Bile Acid-inducible 7α-dehydroxylation Genes in the Human Gut. Comput Struct Biotechnol J. (2019) 17:1016–19. doi: 10.1016/j.csbj.2019.07.012 31428294 PMC6692061

[36] Funabashi M Grove TL Wang M Varma Y McFadden ME Brown LC . A metabolic pathway for bile acid dehydroxylation by the gut microbiome. Nature. (2020) 582:566–70. doi: 10.1038/s41586-020-2396-4 32555455 PMC7319900

[37] Guzior DV Okros M Shivel M Armwald B Bridges C Fu Y . Bile salt hydrolase acyltransferase activity expands bile acid diversity. Nature. (2024) 626:852–58. doi: 10.1038/s41586-024-07017-8 38326608

[38] Jones EV Wang Y Wei W Reed JC Chaudhari SN Li DK . Bile salt hydrolase activity as a rational target for MASLD therapy. Gut Microbes. (2026) 18:2608437. doi: 10.1080/19490976.2025.2608437 41481156 PMC12773562

[39] Shi L Jin L Huang W. Bile Acids, Intestinal Barrier Dysfunction, and Related Diseases. Cells. (2023) 12:1888. doi: 10.3390/cells12141888 37508557 PMC10377837

[40] Gillard J Roumain M Picalausa C Thibaut MM Clerbaux LA Tailleux A . A gut microbiota-independent mechanism shapes the bile acid pool in mice with MASH. JHEP Rep. (2024) 6:101148. doi: 10.1016/j.jhepr.2024.101148 39741697 PMC11686050

[41] Hang S Paik D Yao L Kim E Trinath J Lu J . Bile acid metabolites control T(H)17 and T(reg) cell differentiation. Nature. (2019) 576:143–48. doi: 10.1038/s41586-019-1785-z 31776512 PMC6949019

[42] Song X Sun X Oh SF Wu M Zhang Y Zheng W . Microbial bile acid metabolites modulate gut RORgamma(+) regulatory T cell homeostasis. Nature. (2020) 577:410–15. doi: 10.1038/s41586-019-1865-0 31875848 PMC7274525

[43] Nie Q Luo X Wang K Ding Y Jia S Zhao Q . Gut symbionts alleviate MASH through a secondary bile acid biosynthetic pathway. Cell. (2024) 187:2717–34 doi: 10.1016/j.cell.2024.03.034 38653239

[44] Benede-Ubieto R Cubero FJ Nevzorova YA. Breaking the barriers: the role of gut homeostasis in metabolic-associated steatotic liver disease (MASLD). Gut Microbes. (2024) 16:2331460. doi: 10.1080/19490976.2024.2331460 38512763 PMC10962615

[45] Neumann M Steimle A Grant ET Wolter M Parrish A Willieme S . Deprivation of dietary fiber in specific-pathogen-free mice promotes susceptibility to the intestinal mucosal pathogen Citrobacter rodentium. Gut Microbes. (2021) 13:1966263. doi: 10.1080/19490976.2021.1966263 34530674 PMC8451455

[46] Martin-Grau M Monleon D. The Role of microbiota-related co-metabolites in MASLD progression: a narrative review. Curr Issues Mol Biol. (2024) 46:6377–89. doi: 10.3390/cimb46070381 39057023 PMC11276081

[47] Li DK Chaudhari SN Lee Y Sojoodi M Adhikari AA Zukerberg L . Inhibition of microbial deconjugation of micellar bile acids protects against intestinal permeability and liver injury. Sci Adv. (2022) 8:eabo2794. doi: 10.1126/sciadv.abo2794 36026454 PMC9417178

[48] Yang B Luo W Wang M Tang Y Zhu W Jin L . Macrophage-specific MyD88 deletion and pharmacological inhibition prevents liver damage in non-alcoholic fatty liver disease via reducing inflammatory response. Biochim Biophys Acta Mol Basis Dis. (2022) 1868:166480. doi: 10.1016/j.bbadis.2022.166480 35811033

[49] Kim H Nelson P Nzabarushimana E Shen J Jensen J Bhosle A . Multi-omic analysis reveals transkingdom gut dysbiosis in metabolic dysfunction-associated steatotic liver disease. Nat Metab. (2025) 7:1476–92. doi: 10.1038/s42255-025-01318-6 40604156 PMC12320953

[50] Tokuhara D. Role of the Gut Microbiota in Regulating Non-alcoholic Fatty Liver Disease in Children and Adolescents. Front Nutr. (2021) 8:700058. doi: 10.3389/fnut.2021.700058 34250000 PMC8267179

[51] Xia Y Yang J Lu S Cheng W Ren M Liu Z . Microbial changes resulting from VSG attenuate MASLD by modulating bile acid metabolism and the intestinal FXR-FGF19 axis. mSystems. (2025) 10:e0063425. doi: 10.1128/msystems.00634-25 41114583 PMC12625774

[52] Fiorucci S Carino A Baldoni M Santucci L Costanzi E Graziosi L . Bile acid signaling in inflammatory bowel diseases. Dig Dis Sci. (2021) 66:674–93. doi: 10.1007/s10620-020-06715-3 33289902 PMC7935738

[53] Zheng C Wang L Zou T Lian S Luo J Lu Y . Ileitis promotes MASLD progression via bile acid modulation and enhanced TGR5 signaling in ileal CD8(+) T cells. J Hepatol. (2024) 80:764–77. doi: 10.1016/j.jhep.2023.12.024 38181823

[54] Lai J Luo L Zhou T Feng X Ye J Zhong B. Alterations in circulating bile acids in metabolic dysfunction-associated steatotic liver disease: a systematic review and meta-analysis. Biomolecules. (2023) 13:1356. doi: 10.3390/biom13091356 37759756 PMC10526305

[55] Munte E Hartmann P. The role of short-chain fatty acids in metabolic dysfunction-associated steatotic liver disease and other metabolic diseases. Biomolecules. (2025) 15:469. doi: 10.3390/biom15040469 40305160 PMC12025087

[56] Lian S Lu M Jiajing L Zhang B Fang Y Wang X . Conjugated lithocholic acid activates hepatic tgr5 to promote lipotoxicity and MASLD-MASH transition by disrupting carnitine biosynthesis. Adv Sci. (2025) 12:e2410602. doi: 10.1002/advs.202410602 40344326 PMC12120702

[57] Daemen S Gainullina A Kalugotla G He L Chan MM Beals JW . Dynamic shifts in the composition of resident and recruited macrophages influence tissue remodeling in NASH. Cell Rep. (2021) 34:108626. doi: 10.1016/j.celrep.2020.108626 33440159 PMC7877246

[58] Seidman JS Troutman TD Sakai M Gola A Spann NJ Bennett H . Niche-specific reprogramming of epigenetic landscapes drives myeloid cell diversity in nonalcoholic steatohepatitis. Immunity. (2020) 52:1057–74. doi: 10.1016/j.immuni.2020.04.001 32362324 PMC7305990

[59] Zhang J Wang Y Fan M Guan Y Zhang W Huang F . Reactive oxygen species regulation by NCF1 governs ferroptosis susceptibility of Kupffer cells to MASH. Cell Metab. (2024) 36:1745–63. doi: 10.1016/j.cmet.2024.05.008 38851189

[60] Kim HY Rosenthal SB Liu X Miciano C Hou X Miller M . Multi-modal analysis of human hepatic stellate cells identifies novel therapeutic targets for metabolic dysfunction-associated steatotic liver disease. J Hepatol. (2025) 82:882–97. doi: 10.1016/j.jhep.2024.10.044 39522884 PMC12306656

[61] Li JZ Yang L Xiao MX Li N Huang X Ye LH . Spatial and single-cell transcriptomics reveals the regional division of the spatial structure of MASH fibrosis. Liver Int. (2025) 45:e16125. doi: 10.1111/liv.16125 39400982 PMC11891380

[62] Guilliams M Bonnardel J Haest B Vanderborght B Wagner C Remmerie A . Spatial proteogenomics reveals distinct and evolutionarily conserved hepatic macrophage niches. Cell. (2022) 185:379–96 doi: 10.1016/j.cell.2021.12.018 35021063 PMC8809252

[63] Ramachandran P Dobie R Wilson-Kanamori JR Dora EF Henderson BEP Luu NT . Resolving the fibrotic niche of human liver cirrhosis at single-cell level. Nature. (2019) 575:512–18. doi: 10.1038/s41586-019-1631-3 31597160 PMC6876711

[64] Fallowfield JA Mizuno M Kendall TJ Constandinou CM Benyon RC Duffield JS . Scar-associated macrophages are a major source of hepatic matrix metalloproteinase-13 and facilitate the resolution of murine hepatic fibrosis. J Immunol. (2007) 178:5288–95. doi: 10.4049/jimmunol.178.8.5288 17404313

[65] Li Z Luo G Gan C Zhang H Li L Zhang X . Spatially resolved multi-omics of human metabolic dysfunction-associated steatotic liver disease. Nat Genet. (2025) 57:3112–25. doi: 10.1038/s41588-025-02407-8 41286103 PMC12695644

[66] Watson BR Paul B Rahman RU Amir-Zilberstein L Segerstolpe A Epstein ET . Spatial transcriptomics of healthy and fibrotic human liver at single-cell resolution. Nat Commun. (2025) 16:319. doi: 10.1038/s41467-024-55325-4 39747812 PMC11697218

[67] Wen W Liu Z Tan W Tan Y Li W Wan J . Integrating multi-omics and machine learning systematically deciphers cellular heterogeneity and fibrotic regulatory networks in the progression from MASLD to MASH. NPJ Digit Med. (2026) 9:167. doi: 10.1038/s41746-026-02352-8 41545636 PMC12913776

[68] Li K Kumar V To T Gonzalez-Dominguez A Huria J Yu M . Scar-associated endothelial-stellate cellular crosstalk drives fibrosis resolution in MASH. Cell Rep. (2026) 45:116915. doi: 10.1016/j.celrep.2025.116915 41581151 PMC13010457

[69] Suo Y Thimme R Bengsch B. Spatial single-cell omics: new insights into liver diseases. Gut. (2026) 75:1248–63. doi: 10.1136/gutjnl-2024-332105 40764061 PMC13217129

[70] Harrison SA Loomba R Dubourg J Ratziu V Noureddin M. Clinical Trial Landscape in NASH. Clin Gastroenterol Hepatol. (2023) 21:2001–14. doi: 10.1016/j.cgh.2023.03.041 37059159

[71] Kamrul-Hasan A Mondal S Nagendra L Sasikanth T Ahammed A Rahman S . Role of obeticholic acid, a farnesoid x receptor agonist, in nonalcoholic fatty liver disease: a systematic review and meta-analysis. touchREV Endocrinol. (2024) 20:54–61. doi: 10.17925/EE.2024.20.2.8 39526049 PMC11548348

[72] Sanyal AJ Ratziu V Loomba R Anstee QM Kowdley KV Rinella ME . Results from a new efficacy and safety analysis of the REGENERATE trial of obeticholic acid for treatment of pre-cirrhotic fibrosis due to non-alcoholic steatohepatitis. J Hepatol. (2023) 79:1110–20. doi: 10.1016/j.jhep.2023.07.014 37517454

[73] Harrison SA Abdelmalek MF Neff G Gunn N Guy CD Alkhouri N . Aldafermin in patients with non-alcoholic steatohepatitis (ALPINE 2/3): a randomised, double-blind, placebo-controlled, phase 2b trial. Lancet Gastroenterol Hepatol. (2022) 7:603–16. doi: 10.1016/S2468-1253(22)00017-6 35325622

[74] Harrison SA Neff G Guy CD Bashir MR Paredes AH Frias JP . Efficacy and safety of aldafermin, an engineered FGF19 analog, in a randomized, double-blind, placebo-controlled trial of patients with nonalcoholic steatohepatitis. Gastroenterology. (2021) 160:219–31. doi: 10.1053/j.gastro.2020.08.004 32781086

[75] Harrison SA Bedossa P Guy CD Schattenberg JM Loomba R Taub R . A Phase 3, Randomized, Controlled Trial of Resmetirom in NASH with Liver Fibrosis. N Engl J Med. (2024) 390:497–509. doi: 10.1056/NEJMoa2309000 38324483

[76] Sanyal AJ Newsome PN Kliers I Østergaard LH Long MT Kjær MS . Phase 3 Trial of Semaglutide in Metabolic Dysfunction-Associated Steatohepatitis. N Engl J Med. (2025) 392:2089–99. doi: 10.1056/NEJMoa2413258 40305708

[77] Sanyal AJ Bedossa P Fraessdorf M Neff GW Lawitz E Bugianesi E . A phase 2 randomized trial of survodutide in MASH and fibrosis. N Engl J Med. (2024) 391:311–19. doi: 10.1056/NEJMoa2401755 38847460

[78] Loomba R Sanyal AJ Kowdley KV Bhatt DL Alkhouri N Frias JP . Randomized, controlled trial of the FGF21 analogue pegozafermin in NASH. N Engl J Med. (2023) 389:998–1008. doi: 10.1056/NEJMoa2304286 37356033 PMC10718287

[79] Loomba R Abdelmalek MF Armstrong MJ Jara M Kjaer MS Krarup N. et al. Semaglutide 24 mg once weekly in patients with non-alcoholic steatohepatitis-related cirrhosis: a randomised, placebo-controlled phase 2 trial. Lancet Gastroenterol Hepatol. (2023) 8:511–22. doi: 10.1016/S2468-1253(23)00068-7 36934740 PMC10792518

[80] Newsome PN Buchholtz K Cusi K Linder M Okanoue T Ratziu V . A placebo-controlled trial of subcutaneous semaglutide in nonalcoholic steatohepatitis. N Engl J Med. (2021) 384:1113–24. doi: 10.1056/NEJMoa2028395 33185364

[81] Jiang C Xie C Li F Zhang L Nichols RG Krausz KW . Intestinal farnesoid X receptor signaling promotes nonalcoholic fatty liver disease. J Clin Invest. (2015) 125:386–402. doi: 10.1172/JCI76738 25500885 PMC4382255

[82] Kasai K Igarashi N Tada Y Kani K Takano S Yanagibashi T . Impact of vancomycin treatment and gut microbiota on bile acid metabolism and the development of non-alcoholic steatohepatitis in mice. Int J Mol Sci. (2023) 24:4050. doi: 10.3390/ijms24044050 36835461 PMC9967260

[83] Yang M Zheng X Fan J Cheng W Yan TM Lai Y . Antibiotic-induced gut microbiota dysbiosis modulates host transcriptome and MA epitranscriptome via bile acid metabolism. Adv Sci. (2024) 11:e2307981. doi: 10.1002/advs.202307981 38713722 PMC11267274

[84] Kaufmann B Seyfried N Hartmann D Hartmann P. Probiotics, prebiotics, and synbiotics in nonalcoholic fatty liver disease and alcohol-associated liver disease. Am J Physiol Gastrointest Liver Physiol. (2023) 325:G42–g61. doi: 10.1152/ajpgi.00017.2023 37129252 PMC10312326

[85] Yoon SJ Yu JS Min BH Gupta H Won SM Park HJ . Bifidobacterium-derived short-chain fatty acids and indole compounds attenuate nonalcoholic fatty liver disease by modulating gut-liver axis. Front Microbiol. (2023) 14:1129904. doi: 10.3389/fmicb.2023.1129904 36937300 PMC10014915

[86] Won SM Joung H Park IG Han SH Ham YL Han JS . The effects of next generation probiotics on metabolic dysfunction-associated steatotic liver disease: a parallel, double-blind, randomized, placebo-controlled trial. J Transl Med. (2025) 24:61. doi: 10.1186/s12967-025-07478-z 41366779 PMC12801763

[87] Moszak M Kaczmarczyk M Kregielska-Narożna M Łoniewski I Skonieczna-Żydecka K Szulińska M . Effects of multistrain probiotic supplementation on hepatic function and anthropometric parameters in patients with metabolic dysfunction-associated steatotic liver disease: a double-blind, randomized controlled trial. Nutrition. (2026) 151:113325. doi: 10.1016/j.nut.2026.113325 42456558

[88] Loomba R Hartman ML Lawitz EJ Vuppalanchi R Boursier J Bugianesi E . Tirzepatide for metabolic dysfunction-associated steatohepatitis with liver fibrosis. N Engl J Med. (2024) 391:299–310. doi: 10.1056/NEJMoa2401943 38856224

[89] Francque SM Bedossa P Ratziu V Anstee QM Bugianesi E Sanyal AJ . A randomized, controlled trial of the Pan-PPAR agonist lanifibranor in NASH. N Engl J Med. (2021) 385:1547–58. doi: 10.1056/NEJMoa2036205 34670042

[90] Lun W Yan Q Guo X Zhou M Bai Y He J . Mechanism of action of the bile acid receptor TGR5 in obesity. Acta Pharm Sin B. (2024) 14:468–91. doi: 10.1016/j.apsb.2023.11.011 38322325 PMC10840437

[91] Chen Y Chaudhari SN Harris DA Roberts CF Moscalu A Mathur V . A small intestinal bile acid modulates the gut microbiome to improve host metabolic phenotypes following bariatric surgery. Cell Host Microbe. (2024) 32:1315–30 e5. doi: 10.1016/j.chom.2024.06.014 39043190 PMC11332993

[92] Ramachandran P Brice M Sutherland EF Hoy AM Papachristoforou E Jia L . Aberrant basement membrane production by HSCs in MASLD is attenuated by the bile acid analog INT-767. Hepatol Commun. (2024) 8. doi: 10.1097/HC9.0000000000000574 39585303 PMC11596521

[93] Forlano R Martinez-Gili L Takis P Miguens-Blanco J Liu T Triantafyllou E . Disruption of gut barrier integrity and host-microbiome interactions underlie MASLD severity in patients with type-2 diabetes mellitus. Gut Microbes. (2024) 16:2304157. doi: 10.1080/19490976.2024.2304157 38235661 PMC10798360

